# Effects of Dexamethasone on Remodeling of the Hippocampal Synaptic Filamentous Actin Cytoskeleton in a Model of Pilocarpine-induced Status Epilepticus

**DOI:** 10.7150/ijms.44927

**Published:** 2020-07-02

**Authors:** Nuo Yang, Yan Zhang, Jiang-Tao Wang, Chen Chen, Yan Song, Jian-Min Liang, Di-Hui Ma, Yan-Feng Zhang

**Affiliations:** 1 Department of Pediatric Neurology, The First Hospital of Jilin University, Changchun, Jilin Province 130021, PR China.; 2 Department of Neurology, The First Hospital of Jilin University, Changchun, Jilin Province 130021, PR China.; 3 College of Life Sciences, Jilin University; Jilin Province, 130021, PR China.; 4 Nursing College, Beihua University, 3999 Huashan Road, Jilin 132013, PR China.

**Keywords:** Filamentous actin, Status epilepticus, Dexamethasone, Glucocorticoid receptors, Epileptogenesis, Synapse.

## Abstract

The filamentous actin (F-actin) cytoskeleton is progressively damaged after status epilepticus (SE), which is related to delayed neuronal death, aberrant recurrent circuits and epileptogenesis. Glucocorticoids regulate dendritic spine remodeling by acting on glucocorticoid receptors and the dynamics of the F-actin cytoskeleton. Our previous study showed that administration of dexamethasone (DEX) in the latent period of the pilocarpine epileptic model reduces damage to the hippocampal filamentous actin cytoskeleton and the loss of hippocampal neurons and aids in maintaining the synaptic structures, but it is not sufficient to stop epileptogenesis. In this work, we focused on the role of glucocorticoids in regulating the hippocampal F-actin cytoskeleton during SE. We examined the abundance of synaptic F-actin, analyzed the hippocampal F-actin/G-actin (F/G) ratio and pCofilin, and evaluated the number of hippocampal neurons and pre/postsynaptic markers in pilocarpine-induced status epilepticus mice with or without administration of dexamethasone (DEX). We found that the latency of Stage 3 seizures increased, the mortality decreased, the damage to the synaptic F-actin cytoskeleton in the hippocampal subfields was significantly attenuated, and a greater number of postsynaptic structures were retained in the hippocampal subfields after treatment with DEX. These results indicate that treatment with dexamethasone stabilizes the synaptic F-actin cytoskeleton and reduces the damage to the brain due to SE. This approach is expected to be beneficial in alleviating delayed neuron damage and the process of epileptogenesis.

## Introduction

SE is manifested by the continuous and persistent onset of seizures, which is a life-threatening neurological condition. One of the consequences of SE is extensive brain damage and secondary temporal lobe epilepsy with recurrent spontaneous seizures and hippocampal-dependent cognitive impairment 1, 2. It has been widely reported that acute depolymerization of F-actin in the brain occurs after SE, resulting in a significant reduction in the number and size of dendritic spines 3-5. In addition, delayed and irreversible remodeling of the synaptic actin cytoskeleton occurs following acute F-actin depolymerization, which is related to the delayed death of neurons during the latent period of epileptogenesis 6, 7. The delayed synaptic F-actin cytoskeleton damage is generally extensive and progressive, which is consistent with the delayed and progressive death of neurons in the spatiotemporal distribution 7 Therefore, protecting F-actin from depolymerization during SE is assumed to be beneficial to avoiding the delayed deconstruction of the neuronal actin cytoskeleton and the delayed neuronal death.

Glucocorticoids have proven to be helpful in treatment of epilepsy in numerous clinical and experimental studies 8-12. Glucocorticoids have two type of receptors *in vivo*: mineralocorticoid receptors (MRs) of high affinity and glucocorticoid receptors (GRs) of low affinity 13. Seizure activities can be exacerbated by activating MRs but can be alleviated by regulating synaptic plasticity via activation of GRs in a KA-induced epileptic model 14. Synaptic GRs play important roles in synaptic physiological function and activity-dependent plasticity 15-18. Dynamic changes in the actin network are the main driving forces of synaptic plasticity in terms of structure and function 19. Coincidentally, GRs regulate the morphology and stability of dendritic spines by controlling the dynamic balance of the actin cytoskeleton between depolymerization and polymerization 18. Our previous study showed that administration of dexamethasone during the latent period reduces damage to the hippocampal filamentous actin cytoskeleton and pyramidal neurons and helps to maintain the synaptic structures but is not sufficient to stop epileptogenesis in a pilocarpine-induced epileptic model 20. However, it is still unclear how glucocorticoids affect the process of epileptogenesis by regulating the dynamics of the actin cytoskeleton during SE. In this work, we focused on the role of glucocorticoids in regulating the hippocampal F-actin cytoskeleton in a pilocarpine-induced SE model. After treatment with dexamethasone, we detected changes in seizures and more stabilized synaptic filamentous actin cytoskeleton and synaptic structures. We supply further evidence for the role of GRs activation in controlling the synaptic actin dynamics in the epileptic brain.

## Material and Methods

### Animals

The study protocol for animals was approved by the Research Ethics Committee of the First Hospital of Jilin University, China (reference number 2014-044). The experiments were designed using the principle of "The Three Rs". All experiments were performed on ICR adult male mice weighing 22-24 g. Mice were purchased from Changsheng Biotechnology Co., Ltd. (China, BX). Male animals were chosen to prevent differences in epileptic susceptibility and basal hormone levels due to gender. Mice were housed in quiet rooms with temperature controlled at 22-26 °C with a 12/12 light and dark cycle and were allowed to freely access food and water. Animals were acclimated for 3 days before performing any experiments. To reduce the influence of circadian rhythm on seizures, all pilocarpine-induced SE models were performed between 8 AM and 10 AM.

### Pilocarpine-induced SE

In the morning of the experimental day, mice were injected intraperitoneally with 1 mg/kg methylscopolamine (0.1 mg/ml) (Sigma-Aldrich, MO, USA), followed by 300 mg/kg pilocarpine (30 mg/ml) (Sigma-Aldrich, MO, USA) 30 minutes later. After pilocarpine administration, the behaviors of the animals were monitored for 2 hours, and the onset of seizures was evaluated by Racine scale 21, 22 (1 = mouth and facial movement; 2 = head nodding; 3 = forelimb clonus; 4 = clonic rearing; 5 = rearing and falling). Stage 1, 2 seizures are subtle and prone to observation errors. Therefore, only Stage 3- 5 seizures were recorded and analyzed in this study. The latency of Stage 3 seizures (the period from injection of pilocarpine to the first seizure greater than or equal to Stage 3) was observed, the number of seizures greater than or equal to Stage 3 was counted, and the average seizure stage of each animal was calculated. Two hours after onset, all of the animals received 4 mg/kg diazepam (1 mg/ml) intraperitoneally. After induction of SE, moisture food was supplied to the animals to replenish body fluid.

### Drug Administration and Group Assignment

To observe the effects of glucocorticoids on remodeling of the hippocampal F-actin cytoskeleton during pilocarpine-induced SE, animals were randomly divided into three groups: control, pilocarpine, and pilocarpine with dexamethasone treatment, which were identified as Control, PILO and PILO+DEX groups respectively. For animals in the PILO+DEX group, a dose of 10 mg/kg dexamethasone (diluted in normal saline, 1 mg/ml) (Suicheng Pharmaceutical Co., Ltd, China) was injected intraperitoneally together with pilocarpine.

To verify whether glucocorticoids modulate F-actin dynamics through GRs, pretreatment of RU486, an antagonist of GRs, was administered. In this assay, animals were randomly divided into four groups: control, PILO, PILO+DEX and RU486. For animals in the RU486 group, RU486 (2 mg/ml) (Sigma-Aldrich, MO, USA) was pretreated at a dose of 20 mg/kg for 4 consecutive days intragastrically prior to pilocarpine and dexamethasone administration. The last dose of RU486 was given 1 hour before the pilocarpine and dexamethasone injection. The other three groups were given equal volumes of normal saline intragastrically.

### Sample Preparations

Preparation of samples was performed according to a previously published method 6, 23. One hour after the stop of seizures, the animals were anesthetized and sacrificed, followed by 4% paraformaldehyde perfusion. The brains were removed, immersed in 4% paraformaldehyde for postfixation, and soaked successively in 10%, 20%, and 30% sucrose. Samples were embedded in embedding agent (Tissue-Tek O.C.T. Compound) and frozen at -80℃. Before experiments, samples were cut into 30-micron coronal sections with a Leica cryostat (GmbH, Germany).

### F-actin Labeling

As mentioned previously 6, 23, we labeled F-actin specifically with phalloidin conjugated to Alexa 488. After a thirty-minute pretreatment with 0.3% Triton X-100, sections were incubated in Alexa 488-labeled phalloidin (1:100; A12379, Molecular Probes) in the dark overnight at 4℃. After washing three times, the sections were mounted with anti-fading mounting medium (Immu-Mount, Thermo Scientific, USA) for confocal scanning.

### Hippocampal Pyramidal Cell Quantification

As described in previous studies, hippocampal pyramidal cells were detected by Anti-NeuN antibody 20, 24. After pretreatment of 10% normal donkey serum with 0.3% Triton X-100 for 30 minutes at room temperature, slices were incubated with rabbit monoclonal anti-NeuN antibody (1:100, ab177487, Abcam, USA) overnight at 4 °C. After three washes, slices were incubated in Alexa 488-labeled donkey anti-rabbit antibody (1:200, A21206, Molecular Probes) in the dark for 4 hours at room temperature.

### Labeling of Pre- and Postsynaptic Markers

To test the effect of DEX treatment on presynaptic and postsynaptic components in SE mice, we labeled the presynaptic and postsynaptic structures with synapsin I and PSD95, respectively. Protocols for standard immunofluorescence staining were performed for synapsin I and PSD95 staining. The primary antibodies used were rabbit anti-synaptic polyclonal antibody (1:100; No. 51-5200, Invitrogen, CA, USA) and PSD-95/SAP90 (1:100; No. 51-6900, Invitrogen, CA, USA). For staining of PSD95, pretreatment with pepsin was used to improve the detection 25. Details have been described previously 6, 23.

### Analysis of F/G Ratio and pCofilin

The ratio of F-actin to G-actin reflects the dynamic changes in the actin cytoskeleton in the brain. To measure the F/G ratio, the hippocampi were homogenized, and a standard protocol according to the manufacturer was followed using an Assay KIT (BK037, Cytoskeleton, USA).

The actin-severing protein cofilin is inactivated by Ser3 phosphorylation, thereby allowing further elongation of the polymerizing actin filaments and maintaining the filamentous structures 26. For protein analysis of pCofilin, the hippocampi were homogenized in Cell Extraction Buffer (Molecular Probes) containing Halt™ Protease Inhibitor Cocktail and Halt™ Phosphatase Inhibitor Cocktail (Thermo, Scientific, IL, USA). After centrifugation at 10,000 × g for 10 min, the supernatants were collected, and the protein concentrations were determined and standardized. After sample preparation, a standard protocol for western blot was followed. The primary antibodies included rabbit polyclonal anti-phosphorylated-cofilin (pCofilin) at serine 3 (1:500, ab12866, Abcam, USA) and mouse anti-beta-actin (1:2000, A2228, Sigma Aldrich, MO, USA). For quantification of blot signals, the band intensity was measured with the ImageJ program (National Institutes of Health, USA).

### Image Acquisition and Data Analysis

For confocal scanning and statistical analysis, we randomly selected 7 animals survived from SE in the PILO and PILO+DEX groups and 7 animals from the control group, respectively. A Zeiss 710 confocal laser scanning microscope was used in imaging of F-actin, NeuN, PSD95 and synapse I. Each experiment used the same parameter settings to avoid deviation. Hippocampal slices were randomly selected from the-1.28 to -2.12 mm coordinates of anterior-posterior Bregma. The labeling density of F-actin was detected by Image-Pro Plus analysis software (v. 6.0, Media Cybernetics, Silver Spring, MD). The fluorescence intensity of PSD95 and synapsin I was measured using ImageJ software (National Institutes of Health, USA). We measured at least 3 fields for each hippocampal subregion in each animal. The numbers of pyramidal cells in areas CA1 and CA3 were counted as in the previous description 23, 24, 27. In brief, neurons in the subregions of CA1 and CA3b were counted in a defined rectangular field using ImageJ software. At least 3 sections were selected for each animal.

The raw data were input to GraphPad Prism 8 (GraphPad Software, Inc., La Jolla, CA, U.S.A.) for statistical analysis and graph preparation after the normality test using SPSS25 (IBM Corp., IBM SPSS Statistics Version 25.0, Armonk, NY, U.S.A). Data that followed a normal distribution were expressed as the mean ± SD. One-way ANOVA with the post hoc Tukey's test was performed for data with three or more groups. Data that followed a nonnormal distribution were expressed as M (P25, P75). For comparison between two groups, the Kolmogorov-Smirnov test was used. For conditions among three groups, the Kruskal-Wallis test was performed, followed by the post hoc multiple comparison of Dann's test. The significance set for statistical analysis was P < 0.05.

## Results

### Influence of Dexamethasone Treatment on Acute Seizures Induced by Pilocarpine

After pilocarpine injection, the behavioral changes of animals in this research were similar to those of previous studies 6, 7, 20. As a result, in the PILO and PILO+DEX groups, the ratios of animals that met the SE and survived were 47.62% and 45.83%, and the mortality rates were 42.86% and 25%, respectively. No seizures or deaths were found in the control group.

**Figure 1:**
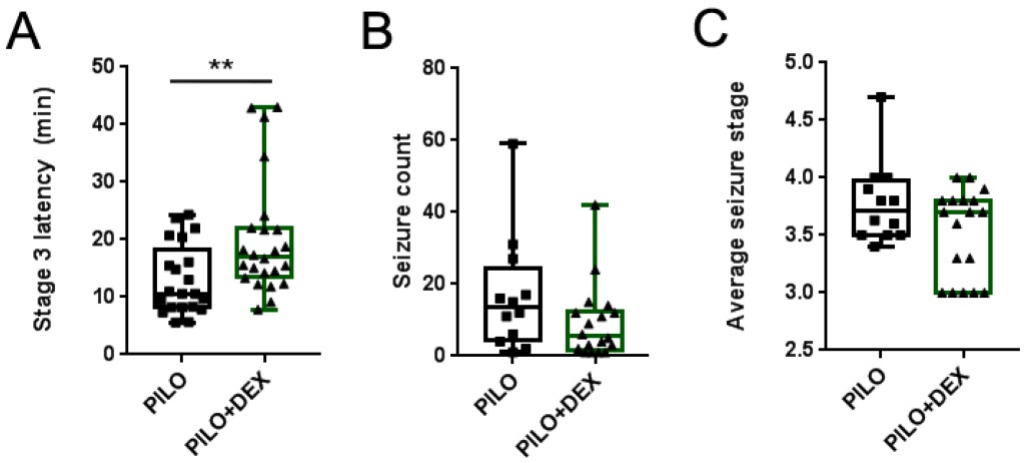
** Influence of acute seizures after treatment with dexamethasone.** A, B and C show the latency of Stage 3 (A), seizure count (B), and average stage of seizures (C). Dexamethasone treatment significantly increased the latency of Stage 3 in pilocarpine-induced acute seizures, but the number and the average stage of acute seizures did not show a significant difference between the two groups (Kolmogorov-Smirnov test; A, n1=21, n2=24; B and C, n1=12, n2=18; **, p<0.01). Data are represented as median (black bar), 25-75 percentile (box), and Min-Max (whiskers).

We observed the latency of Stage 3 seizures in all animals of the PILO and PILO+DEX groups, counted the number of seizures of greater than or equal to Stage 3, and calculated the average stage in the survived animals. The average latencies of Stage 3 seizures were 10.60 (8.20, 18.23) minutes and 16.99 (13.53, 21.93) minutes in the PILO and PILO+DEX groups, respectively. Compared with the PILO group, dexamethasone treatment significantly increased the latency of Stage 3 seizures (P<0.01) (Fig. 1A). However, no difference was found between the two groups in the number and the average stage of seizures (P=0.40, 0.23) (Fig. 1B and C).

### Dexamethasone Treatment Attenuated the Hippocampal F-actin Damage after SE

**Figure 2:**
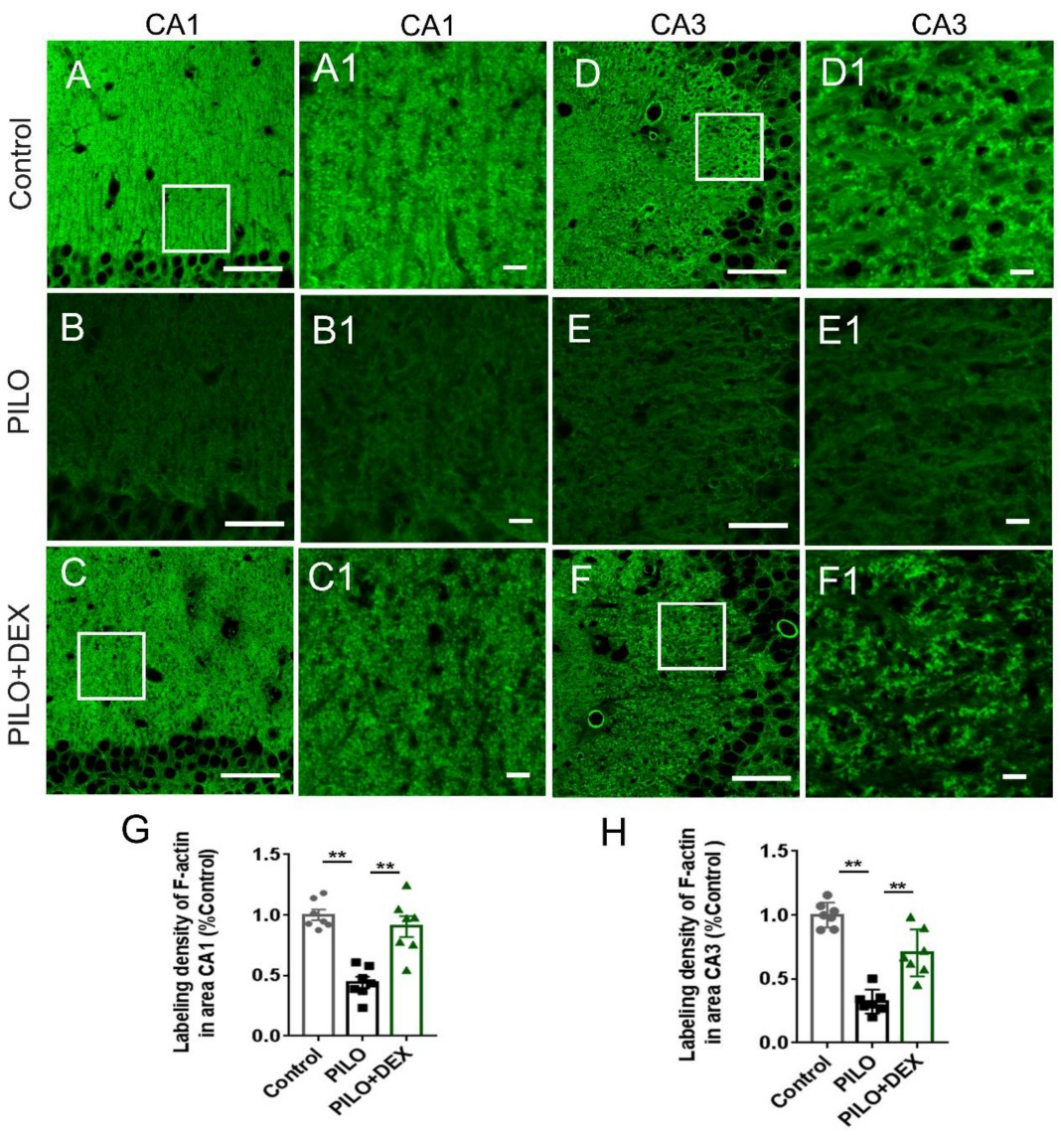
** Dexamethasone treatment alleviates F-actin cytoskeleton damage after pilocarpine-induced SE.** Scale bars: A-F, 50 μm; A1-F1, 5 μm. The labeling density of F-actin in hippocampal subregions CA1 (B and B1, G) and CA3 (E and E1, H) decreases remarkably after SE induced by pilocarpine. Administration of dexamethasone rescues the damage of F-actin to a large extent (C and C1 for CA1, F and F1 for CA3, G and H for statistical analysis) (n=7; One-way ANOVA, **, p<0.01). Data are represented as mean ± SD.

To examine the influence of DEX treatment on actin cytoskeleton dynamics during SE, hippocampal F-actin was detected with phalloidin-specific labeling. Compared with the controls (Fig. 2A-A1 and D-D1), the F-actin intensities in the CA3 and CA1 subregions of the PILO group were reduced by more than 50% and 60% after SE (Fig. 2B-B1 and E-E1). Dexamethasone treatment ameliorated the F-actin reduction to 90.36% and 70.32% of the control, respectively (Fig. 2C-C1 and F-F1). Statistical analysis demonstrated that dexamethasone treatment remarkably reduced the damage of F-actin in the CA3 and CA1 subfields after SE (P<0.01) (Fig. 2G and H).

**Figure 3:**
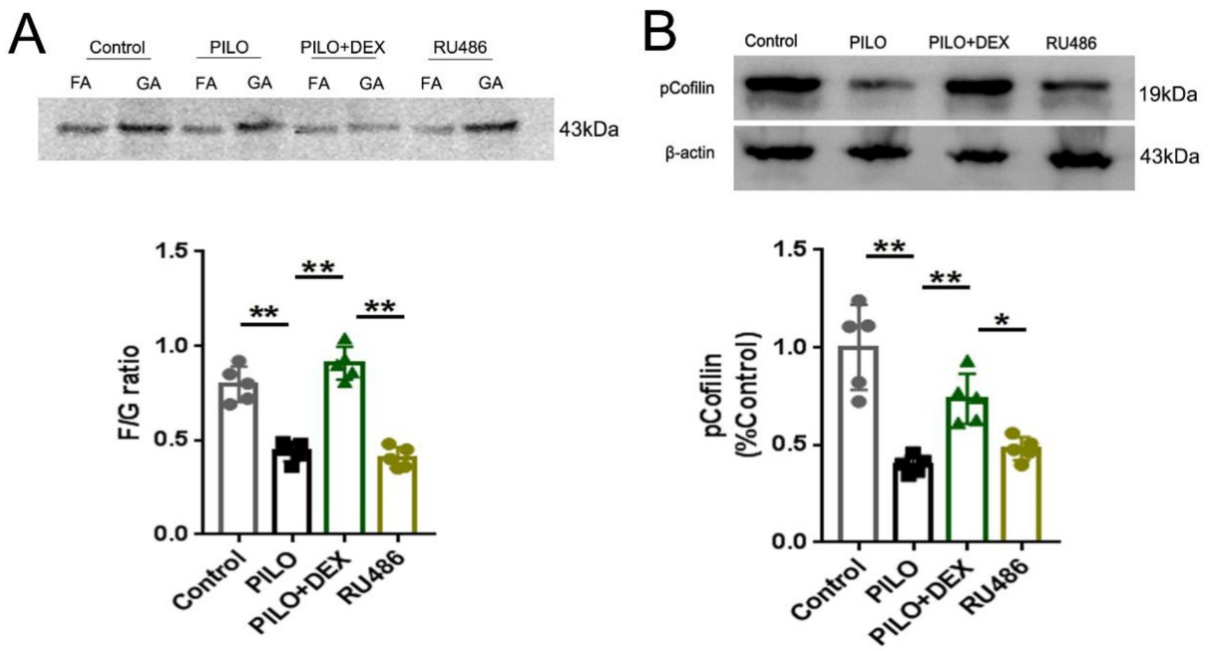
** Analysis of F/G ratio and pCofilin.** A: F/G ratio analysis shows that the F/G ratio is significantly decreased in the PILO group relative to that of the control group. Treatment of DEX reverses this effect. With RU486 pretreatment, the F/G ratio is not restored B: Western blot analysis of pCofilin shows a significantly decreased signal in the PILO group relative to the control group. After treatment of DEX, the signal intensity of pCofilin increases significantly. In the RU486 group, the signal intensity does not recover significantly (n=5; One-way ANOVA, **, p<0.01; *, p<0.05). Data are represented as mean ± SD.

To determine the effect of DEX on F-actin polymerization, we measured the F/G ratio and analyzed the protein pCofilin. Pretreatment of RU486, a GRs antagonist, was applied to further determine whether DEX exerts these effects through GRs. Analysis of the F/G ratio showed that the average F/G ratio was approximately 0.80 in the control group and decreased to 0.44 in the PILO group. Treatment with DEX increased this ratio to 0.91, whereas with RU486 pretreatment, the F/G ratio decreased to 0.41 (Fig. 3A). Statistical analysis demonstrated that dexamethasone treatment prevented the depolymerization of F-actin to G-actin after SE (P<0.01) (Fig. 3A). However, pretreatment of RU486 blocked this effect (P<0.01) (Fig. 3A). Protein analysis of pCofilin showed that the signal in the PILO group was decreased to approximately 40% of the control (P<0.01). After treatment with DEX, the signal intensity of phosphorylated cofilin was increased to 73.61% of the control (P<0.01). However, in the RU486 pretreatment group, the signal intensity did not recover significantly (48.03% of control) (P<0.05), which indicates that glucocorticoids might promote polymerization of the actin skeleton through GRs (Fig. 3B).

### The Number of Pyramidal Neurons Was Unaltered after SE in both Pilocarpine and DEX Treatment Groups

**Figure 4:**
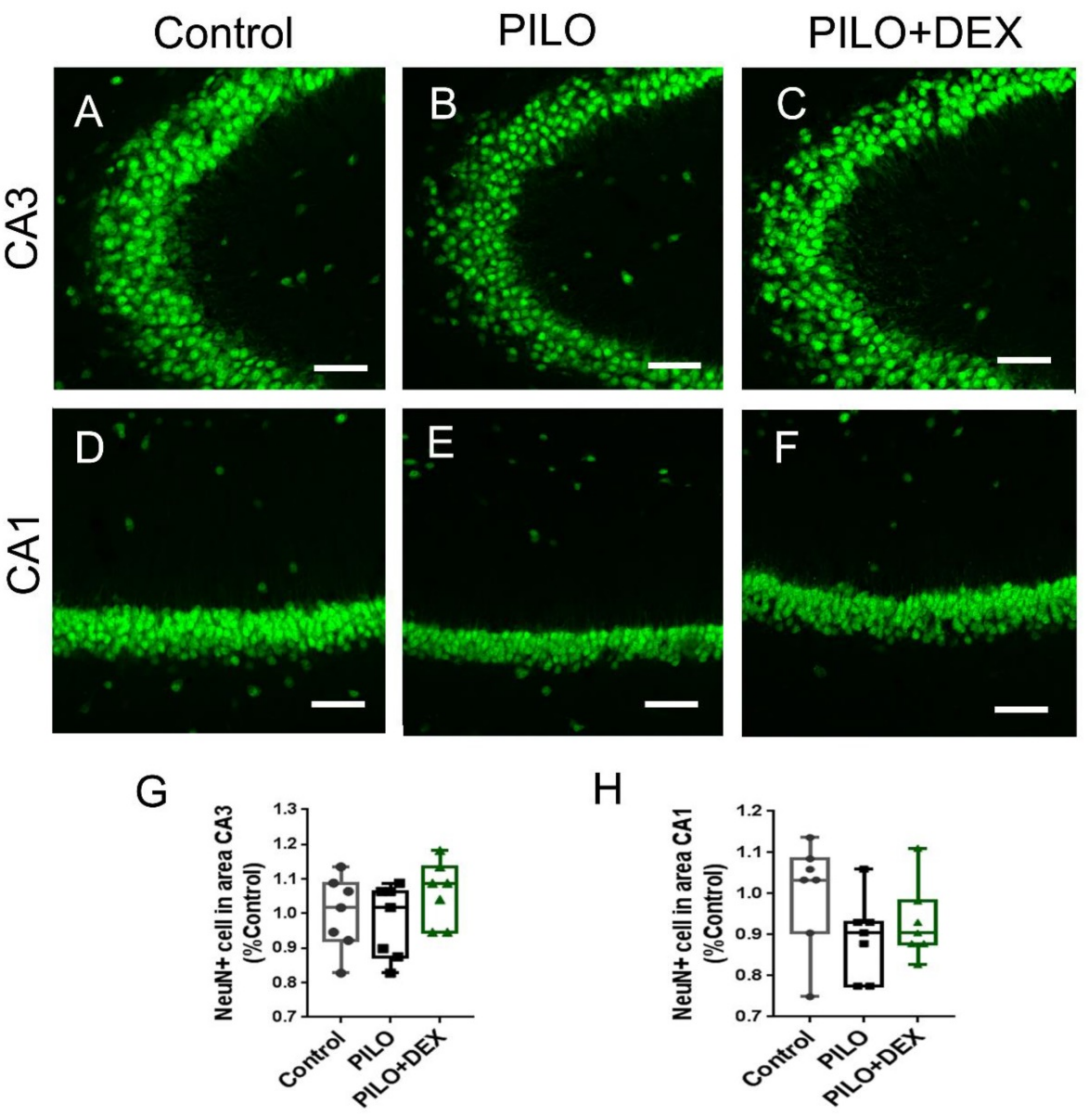
** The number of pyramidal neurons is unaltered after acute seizures induced by pilocarpine and DEX treatment.** Scale bars: 100 μm. The number of pyramidal neurons in subfields CA3 (A, B, C, and G) and CA1 (D, E, F and H) does not change remarkably after SE in both PILO and PILO+DEX groups (Kruskal-Wallis test, n =7). Data are represented as median (black bar), 25-75 percentile (box), and Min-Max (whiskers).

To examine whether pilocarpine-induced SE or dexamethasone treatment had an influence on the seizure-induced neuron damage, anti-NeuN staining was performed. Relative to the controls, the number of pyramidal neurons in subfields CA3 (Fig. 4A, B, C, and G) and CA1 (Fig. 4D, E, F and H) did not change remarkably in both the PILO and PILO+DEX groups (P=0.25, 0.27).

### Effects of DEX Treatment on Synaptic Remodeling after Pilocarpine-induced SE

**Figure 5:**
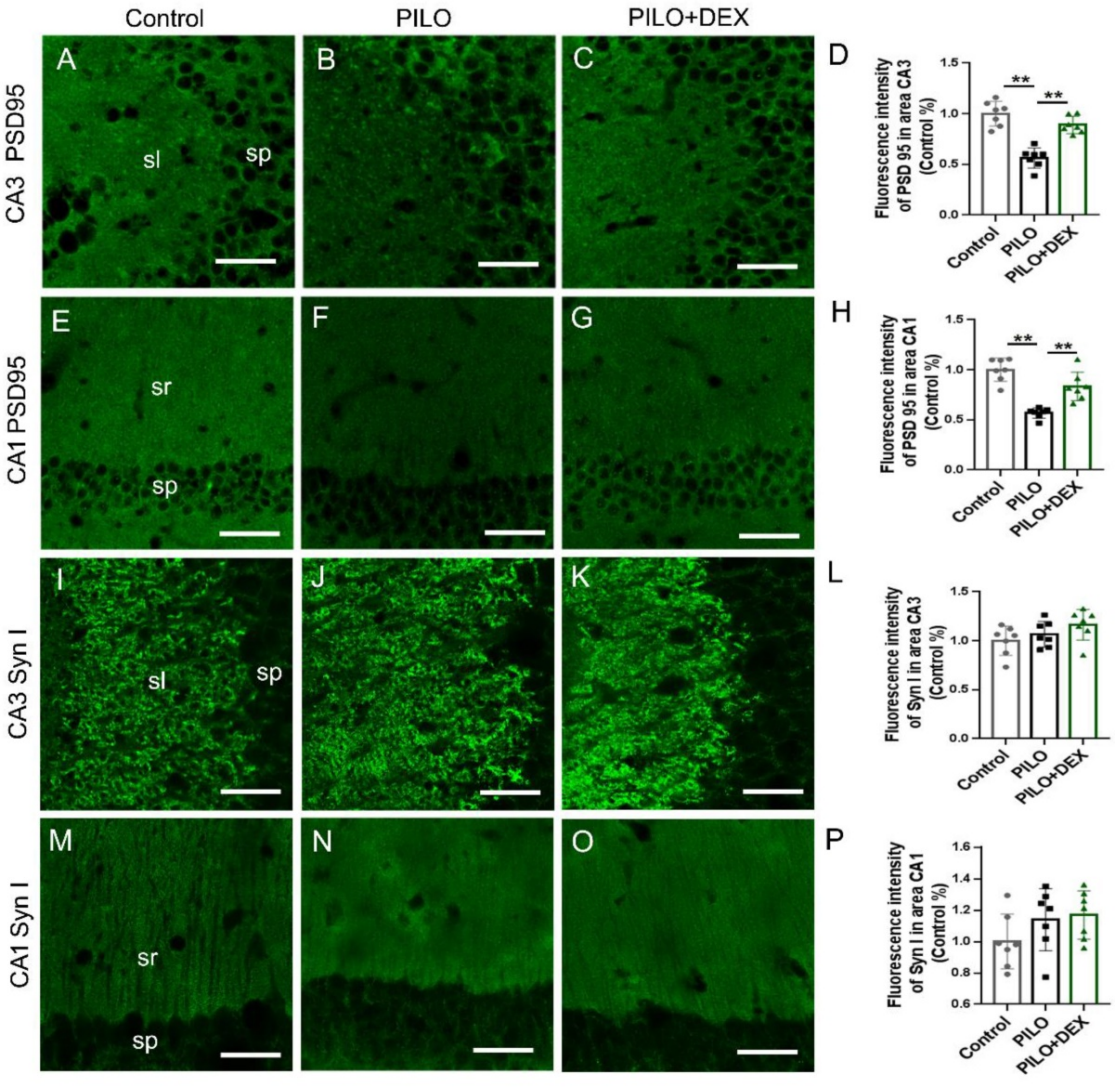
** Effect of dexamethasone treatment on pre- and postsynaptic markers after pilocarpine-induced SE.** Scale bars: 50 μm. The labeling intensity of PSD95, the postsynaptic marker, decreases remarkably in subfields CA3 (B and D) and CA1 (F and H) after SE induced by pilocarpine. Treatment with dexamethasone reduces the decrease of PSD95 in subfields CA3 (C and D) and CA1 (G and H). The labeling intensity of synapsin I, the presynaptic marker, does not change remarkably in subfields CA3 (I, J, K and L) and CA1 (M, N, O and P) after SE induced by pilocarpine in both PILO and PILO+DEX groups (n=7; One-way ANOVA, **, p<0.01). Data are represented as mean ± SD.

Pre/postsynaptic structures were examined to determine whether dexamethasone treatment affects synaptic remodeling after SE. The labeling intensity of synapsin I, the presynaptic marker, did not change remarkably in the subfields CA3 (P=0.14) (Fig. 5I, J, K, and L) and CA1 (P=0.20) (Fig. 5M, N, O and P) after SE in both the PILO and PILO+DEX groups. The labeling intensity of PSD95, the postsynaptic marker, was reduced to 56.35% and 56.79% of that in the control in the subfields CA3 (Fig. 5B) and CA1 (Fig. 5F) in the PILO group, and after dexamethasone treatment it was recovered to 88.95% and 83.44% respectively (Fig. 5C and G). Statistical analysis confirmed that the labeling intensity of PSD95 decreased remarkably in subfields CA3 (P<0.01) (Fig. 5D) and CA1 (P<0.01) (Fig. 5H) after SE. Dexamethasone treatment mitigated the decrease of PSD95 in areas CA3 (P<0.01) (Fig. 5D) and CA1(P<0.01) (Fig. 5H).

## Discussion

Numerous studies have focused on the effects of glucocorticoids in epilepsy and convulsions, but the conclusions are controversial so far 11, 12, 28-33. Different dosages, courses, and time points of glucocorticoid administration might be responsible for these discrepancies. The influence of different dosages of dexamethasone were evaluated in epileptic models. In a lithium-pilocarpine-induced model, three doses of DEX (5, 10, and 20 mg/kg) were checked, and only DEX at a dose of 10 mg/kg demonstrated an effect of seizure reduction 29. In a penicillin-induced model, DEX at dosages of 1, 3, and 10 mg/kg were assessed on epileptiform activity, and spike frequencies were significantly suppressed by DEX administration at both 3 mg/kg and 10 mg/kg 30. Consistently, our previous study also showed a protective effect on the epileptic brain with 3 doses of 10 mg/kg DEX during quiescence in the pilocarpine-induced epileptic model 20. In this study, we performed administration of DEX at a different time point to test the effect of DEX during SE and further studied the effects of glucocorticoids on brain remodeling. In line with previous findings, our results showed that DEX at 10 mg/kg displayed a prolonged incubation of Stage 3 seizures and decreased mortality during pilocarpine-induced SE.

MRs and GRs are two types of glucocorticoid receptors in the brain. High-dose glucocorticoids activate GRs, and low-dose glucocorticoids activate MRs. However, the activation of these two types of glucocorticoid receptors often plays opposite roles in many physiological activities 34, 35. The inverse activity of MRs and GRs is a reasonable interpretation for the different results of glucocorticoids in treatment of epilepsy due to different doses. Therefore, we hypothesized that GRs were activated with the administration of 10 mg/kg of DEX in our studies.

Dexamethasone treatment showed a protective effect on the hippocampal F-actin cytoskeleton in this study. Numerous studies have shown that glucocorticoids regulate the F-actin cytoskeleton through two main pathways, i.e., transcriptional and nontranscriptional pathways. The dynamics of the actin cytoskeleton might be affected by transcriptional regulation of glucocorticoid-induced gene expression 36-38. However, the nontranscriptional pathway regulation induced by glucocorticoids also affects actin rearrangement 39-41. GRs are proved to localize in dendritic spines and regulate the actin dynamics through G-protein coupled pathways 18. Administration of the GR agonist dexamethasone rapidly increases the phosphorylation of cofilin, leads to the activation of extracellular signal-regulated kinase (ERK) ½, and accordingly stabilizes the actin cytoskeleton in dendritic spines. The GR antagonist RU486 can blocked this effect 18. This study also confirmed that glucocorticoids can promote the polymerization of F-actin during pilocarpine-induced SE, whereas the administration of RU486 antagonized this effect.

In view of the evidence presented, we inferred that DEX might promote the polymerization of actin and maintain the filamentous state by activating GRs on the dendritic spines in our study.

Many reports have stated that glucocorticoids have a protective effect on neuronal loss in epilepsy models 20, 42. Therefore, we explored the changes in hippocampal neurons among groups. Our data showed that at 1 hour after SE, no significant change in the number of pyramidal neurons in the CA3 and CA1 subregions among the three groups. This result is consistent with our previous study 7. After SE, the number of pyramidal cells in the CA1-CA3 region was observed at 6h, 24h, 2d, 3d, 5d, 7d, and 14d and it showed the damage to the pyramidal neurons was detected from 24h and gradually extended from 2 to 14 days 7. This indicates that the protective effect of DEX on the F-actin cytoskeleton in the present study is achieved through local synaptic effects. However, this result also suggests that this is a highly crucial window and that treatment during this period might reduce or further prevent delayed death of neurons.

Evidence suggests that actin cytoskeleton exerts a key role in the communication network to determine cell death or survival 43. Stabilization of the actin cytoskeleton improves the survival potential to apoptosis, while destabilization of actin cytoskeleton abets cell death 43. Thus, we speculate that preservation of the F-actin structure by DEX treatment in this study might help neurons to survive in the following period.

F-actin is located in both pre- and postsynaptic structures, interacts with pre- and postsynaptic proteins and plays a key role in synaptic remodeling 19, 44-46. Thus, we examined the effect of glucocorticoids on synaptic remodeling of hippocampal neurons after SE. The application of glucocorticoids can rapidly increase the density of the thorny excrescences of the CA3 pyramidal neurons by activating synaptic GRs and downstream kinase-related pathways 47. Consistent with these data, our results showed that relatively more postsynaptic structures were retained in the dexamethasone treatment group after pilocarpine-induced SE, whereas the presynaptic marker did not present corresponding changes. This result indicates that DEX might work primarily through the activation of postsynaptic GRs. The fact that more filamentous structures of actin were preserved in the hippocampus after treatment of glucocorticoid offers further proof of the crucial role of F-actin stabilization in synaptic remodeling 19, 44.

F-actin depolymerization after status epilepticus has been widely known and might be related to subsequent remodeling of synapses and delayed neuronal death. Therefore, protecting the F-actin cytoskeleton from depolymerization during SE might play an important role in blocking the process of epileptogenesis.

## Conclusions

In summary, we examined the remodeling of actin dynamics in the SE model after glucocorticoid treatment. Our results demonstrate that administration of 10 mg/kg dexamethasone ameliorates the damage to the hippocampal filamentous actin cytoskeleton, contributes to the maintenance of synaptic structures, and might play a role in blocking the process of epileptogenesis.
